# DTR-SHIELD: Mutual Synchronization for Protecting against DoS Attacks on the SHIELD Protocol with AES-CTR Mode

**DOI:** 10.3390/s24134163

**Published:** 2024-06-26

**Authors:** Sang-su Lee, Jong-sik Moon, Yong-je Choi, Daewon Kim, Seungkwang Lee

**Affiliations:** 1 Cyber Security Research Division, Electronics and Telecommunications Research Institute, Daejeon 34129, Republic of Koreajsmoon@etri.re.kr (J.-s.M.);; 2 Department of Cyber Security, Dankook University, Yongin 16890, Republic of Korea

**Keywords:** supply chain security, IC counterfeiting, DARPA, SHIELD, dielet, DoS attacks

## Abstract

To enhance security in the semiconductor industry’s globalized production, the Defense Advanced Research Projects Agency (DARPA) proposed an authentication protocol under the Supply Chain Hardware Integrity for Electronics Defense (SHIELD) program. This protocol integrates a secure hardware root-of-trust, known as a dielet, into integrated circuits (ICs). The SHIELD protocol, combined with the Advanced Encryption Standard (AES) in counter mode, named CTR-SHIELD, targets try-and-check attacks. However, CTR-SHIELD is vulnerable to desynchronization attacks on its counter blocks. To counteract this, we introduce the DTR-SHIELD protocol, where DTR stands for double counters. DTR-SHIELD addresses the desynchronization issue by altering the counter incrementation process, which previously solely relied on truncated serial IDs. Our protocol adds a new AES encryption step and requires the dielet to transmit an additional 100 bits, ensuring more robust security through active server involvement and message verification.

## 1. Introduction

Outsourcing integrated circuit (IC) fabrication is a prevalent practice in the semiconductor industry, involving designing, manufacturing, testing, and packaging. This process often sees ICs navigating through various supply chain stages, sometimes beyond the original manufacturers’ control. Such detachment can lead to risks like intellectual property breaches, unauthorized overproduction, reverse engineering, and the introduction of counterfeit ICs.

Counterfeit ICs pose a notable financial and operational risk in the semiconductor sector. These inferior components may not meet the intended specifications, potentially causing electronic equipment to malfunction or fail. This can result in costly repairs or replacements, operational downtime, diminished customer trust, and potential legal challenges if the counterfeit components cause harm or significant disruption. Counterfeit ICs pose a substantial financial threat, particularly in critical sectors like military, aerospace, and medical devices. The industry’s financial loss due to counterfeiting, estimated at USD 7.5 billion annually, underscores the growing concern over counterfeit components in embedded devices [1]. This issue also contributes to significant job losses within the sector [2], highlighting the need for stringent measures to combat semiconductor counterfeiting.

Supply chain security has garnered significant attention within the hardware security community [3,4,5]. Guin and colleagues [6] introduced a taxonomy that categorizes supply chain vulnerabilities into seven distinct types. The first type, known as cloned vulnerability, involves adversaries engaging in illegal activities during the distribution phase, such as copying design files or reverse-engineering chips to reduce IC design costs. The second type, tampered vulnerability, pertains to adversaries inserting hardware Trojans at any point in the supply chain, enabling malicious or destructive behaviors. Overproduced vulnerability, the third type, occurs when foundries or assembly facilities unlawfully sell surplus chips to generate extra profit. The fourth type, defective vulnerability, involves irresponsible manufacturers selling flawed chips misrepresented as fully functional in open markets. Despite the need for electronic component recycling after their useful lifespan, the fifth type, recycled vulnerability, relates to unethical practices where recycled ICs are repackaged and remarked as new. In the sixth type, remarked vulnerability, adversaries remove original markings on chips and apply new coatings to sell them with false, inflated specifications. Lastly, the seventh type of vulnerability, forged documentation, encompasses instances where counterfeiters produce falsified documentation, including forged component revision histories or certifications of compliance with specific standards.

To enhance the security of embedded devices, an additional layer of trust is necessary in the hardware supply chain. Physically unclonable functions (PUFs) have garnered considerable attention as a potential solution [7,8,9,10,11]. PUFs are hardware security primitives that exploit the inherent manufacturing variations in electronic devices to generate unique, unpredictable, and unclonable cryptographic keys or identifiers. Two physically identical devices will exhibit slightly different responses to the same input due to manufacturing variations. This variability can be leveraged to generate a unique and confidential key for each device without the need for storing or transmitting the key.

PUFs offer various applications to enhance the security of the hardware supply chain, encompassing secure boot and firmware verification, anti-counterfeiting measures, key storage and management, and supply chain tracking [12,13]. In secure boot and firmware verification, PUFs play a pivotal role by generating unique keys that validate the integrity of the firmware or boot loader [14,15]. For anti-counterfeiting purposes, PUFs generate distinctive identifiers for each device, which can be verified by the manufacturer or end user, effectively thwarting counterfeit operations [16]. PUFs also excel in key storage and management as they can securely house cryptographic keys without relying on external memory [17,18]. These PUF-generated keys find application in encryption, decryption, and authentication processes. Furthermore, in supply chain tracking, PUFs enable the monitoring of device movement along the supply chain by assigning a unique PUF-generated identifier to each device [11].

However, despite their potential advantages, PUFs come with their fair share of challenges and limitations that need to be addressed for widespread adoption. PUFs depend on inherent manufacturing variability to generate unique responses, which can result in inconsistencies and reduced reliability over time. Mitigation techniques, such as error correction codes and aging compensation, have been proposed to counter this issue [19,20]. Additionally, PUFs often rely on the secrecy of challenge–response pairs (CRPs) to safeguard device security [21,22,23]. However, if an attacker gains access to the CRPs, they can clone the device or generate valid responses without possessing the original device. Various attack vectors, including model-based attacks, side-channel attacks, and invasive attacks, have been proposed to extract CRPs from PUFs. Furthermore, for broad adoption, PUFs need to be scalable and compatible with existing hardware and software architectures. Unfortunately, PUFs often necessitate additional hardware and software support, potentially increasing the overall cost and complexity of the system.

IC packaging can be engineered to resist tampering attempts, but an alternative strategy involves gaining full control over the packaging process to equip ICs with trusted, intelligent, and tamper-evident packages. The Defense Advanced Research Projects Agency (DARPA) takes this concept further with its Supply Chain Hardware Integrity for Electronics Defense (SHIELD) protocol. This protocol proposes integrating a permanent hardware root-of-trust, termed a dielet, within the host package of every legitimately manufactured IC [24].

The dielet possesses passive sensing capabilities, enabling it to detect and record instances of suspicious activities, such as unexpected exposure to light or vibrations. These data can later serve as evidence of potential tampering. An innovative labeling technique known as SHIELD has been developed, seamlessly integrable into existing IC manufacturing and supply chains, ensuring backward compatibility. By incorporating a dielet into the host chip’s packaging, any standard package can be transformed into a tamper-evident one. The authentication system comprises three main components: the dielet itself, inserted into the host chip’s package, a smartphone, and a secure remote server. The smartphone and server establish wireless communication via the network, while the dielet connects to the smartphone through a radio frequency (RF) channel. Beyond providing a unique and permanent identification, the dielet incorporates various sensors capable of measuring parameters like temperature, light exposure, vibration, and ultraviolet (UV) radiation, among others.

Jin and Dijk introduced an enhancement to SHIELD, aptly named CTR-SHIELD [25], which incorporates a counter block in non-volatile memory (NVM) to enable AES counter mode (AES-CTR) encryption [26,27]. They also integrated a true random number generator (TRNG) [28,29] to enable dielets to autonomously generate a serial ID and a secret key simultaneously during a trusted manufacturing process. This initialization process assigns a unique serial ID and a cryptographic key to each dielet.

CTR-SHIELD not only reduces power consumption but also thwarts “try-and-check” attacks while minimizing communication rounds to just one. However, the read-out mode that precedes authentication effectively counters batch-mode denial-of-service (DoS) attacks but leaves single-dielet DoS attacks as a potential vulnerability. By initiating a read-out mode with a specific dielet, an adversary can increment its counter until it reaches its limit. This could be followed by launching batch-mode DoS attacks by applying the same technique to multiple dielets. Even though this attack assumes that the targeted dielets are eventually discarded, it remains a remote threat to legitimate dielets. Consequently, further research is needed to devise comprehensive solutions against single-dielet DoS attacks. This paper proposes an enhancement to CTR-SHIELD to prevent desynchronization of the shared counters between the server and dielets. The primary contributions of this paper are as follows:

- We propose a secure method for achieving a mutual synchronization of the counters shared between the dielet and the server, effectively preventing desynchronization attacks.
- To reduce computational costs for mutual synchronization, we introduce a new counter block in the dielet. In the previous implementation of CTR-SHIELD, the server required up to *T* encryption operations to synchronize the counter blocks. However, applying this approach directly to the dielet would be excessively computationally burdensome. Our approach significantly reduces this overhead to a maximum of two encryption operations. We achieve this by incorporating an additional counter block at the dielet side, effectively tracking the server’s counter block. As a result, the low-cost dielet avoids the need for backtracking the correct value of the desynchronized counter block, eliminating multiple computationally expensive encryptions.
- We offer three crucial corrections or comments to address inaccuracies in the description of CTR-SHIELD [25], ensuring the presentation of correct information. Please find these corrections or comments marked in italics in Section 2.

## 2. Previous Work

DARPA, a research agency of the United States Department of Defense, is focused on developing emerging technologies for military purposes. One of the major concerns for DARPA is the security of the hardware supply chain, which is susceptible to attacks that can compromise the safety and integrity of military systems. To address this concern, DARPA introduced the SHIELD protocol, which incorporates a sequence of authentication and verification measures to protect the hardware supply chain from malicious attacks. However, the authentication protocol developed by DARPA is susceptible to a vulnerability referred to as the “try-and-check” attack. In this attack, an adversary covertly attempts to detach a legitimate dielet and relabel it onto a counterfeit chip without being detected. To address this issue, Jin and Dijk proposed an improved version of the SHIELD protocol called CTR-SHIELD, which incorporates additional security measures to resist “try-and-check” attacks. The CTR-SHIELD protocol uses a counter block to encrypt a challenge, making it more secure than the DARPA’s authentication protocol. Nonetheless, the CTR-SHIELD protocol is still susceptible to desynchronization attacks, where an attacker can disrupt the synchronization between the counter blocks of the chip and the server, causing the authentication process to fail.

### 2.1. Notations

The following notations are used in the overviews of SHIELD and CTR-SHIELD:

- ENC(*K*, *C*) denotes the encryption of a challenge *C* with the key *K* using a block cipher ENC.
- The symbol || represents bit concatenation.
- AES(*K*, C||CB) represents the concatenation of a random nonce *C* and the counter block CB, which is then encrypted with the key *K* using the AES algorithm.
- ${\left[\mathrm{Serial}\;\mathrm{ID}\right]}_{L}$ refers to the truncation of the Serial ID to *L* bits.

### 2.2. DARPA’s Authentication Protocol For Shield

The SHIELD system consists of two main protocols: initialization and authentication. The initialization protocol updates the list of validated dielet serial IDs and their respective secret keys after completing the initialization process. In the trust model, the server is considered trustworthy, and adversaries can only access the list of serial IDs through interaction with the server. Although the serial IDs are unencrypted and visible to adversaries, they are only used to initiate protocol interactions. The server’s database contains other sensitive information, which is strictly for internal use to verify the status and authenticity of the dielets.

**Figure 1:**
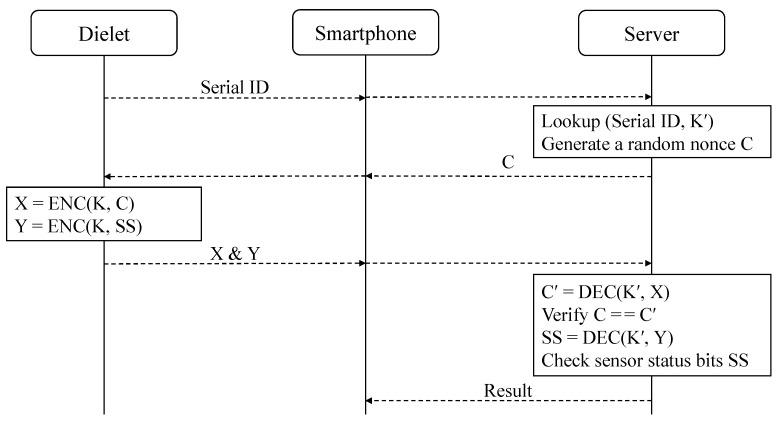
DARPA’s SHIELD authentication protocol consists of two communication rounds, utilizing a 64-bit serial ID, a 128-bit challenge, and two 128-bit ciphertexts. In total, 448 bits are transmitted between the dielet and the smartphone.

The authentication protocol for the SHIELD program, developed by DARPA, is shown in Figure 1. To authenticate the host chip, a smartphone equipped with an inductive or RF probe is utilized. The process begins by powering up the dielet and transmitting its serial ID to the server. The server searches for the corresponding entry in its database. If a match is found, the server sends a random nonce *C* to the dielet via the smartphone. The dielet employs its secret key *K* to encrypt both the nonce *C* and the sensor status bits *SS*, resulting in ciphertexts *X* and *Y*, which are then returned to the server. The server utilizes the key $K^{\prime }$ associated with the stored serial ID to decrypt the ciphertext *X* and compares the resulting $C^{\prime }$ with the original challenge *C*. If $C^{\prime }$ is equal to *C* and no attacks are recorded in the sensor status bits, the server verifies the authenticity of the chip. In the final step, the server communicates the authentication result to the smartphone. To safeguard against man-in-the-middle attacks, it is assumed that a secure communication channel is established between the smartphone and server, incorporating a reliable authentication protocol.

Regrettably, this authentication protocol suffers from a vulnerability known as a “try-and-check” attack, which allows the reuse of dielets that have been detached from legitimate chips. This flaw significantly undermines the effectiveness of the authentication protocol. When a dielet is separated from its legitimate host package and integrated into an illicit or Trojan-infected chip, the passive sensors on the dielet can detect the physical attack with a probability of greater than zero, denoted as ρ. However, an attacker may attempt to insert the dielet into the host package of a malicious chip, assuming the passive sensors will fail to detect the separation with a probability of 1 − ρ. Presently, the attacker can exploit the same challenge to verify if the replied ciphertext remains unchanged. If the ciphertext remains unaltered, the attacker can infer that the passive sensors have not detected the physical attack, which would have led to modifications in the sensor status bits and the ciphertext. Consequently, the attacker can determine which counterfeit chips will successfully pass the authentication protocol. This enables the attacker to identify counterfeit or malicious chips that can be introduced into the supply chain without raising suspicion from the authentication server. By repeating this procedure for all authentic dielets, the “try-and-check” attacker can assemble a collection of successfully separated dielets that can evade detection during authentication.

### 2.3. CTR-SHIELD

Jin and Dijk have proposed an improved version of the SHIELD protocol that aims to counteract the “try-and-check” attack [25]. This protocol adapts the AES-CTR mode, which is approved by the National Security Agency (NSA) Suite B Cryptography and recommended by NIST [26], to prevent challenges from producing identical ciphertext. This protocol is referred to as CTR-SHIELD in this paper. Nevertheless, the counter mode encryption deployed in this protocol possesses a vulnerability that might be leveraged in a DoS attack. In such an attack, an attacker could activate a group of dielets, prompting each one to increment its counter in an irreversible manner. This attack, known as a desynchronization attack, results in two copies of the counter being desynchronized between a dielet and the server. To prevent significant losses, a dielet is required to undergo a read-out mode before entering the authentication mode. This mode guarantees that the current message is accurately delivered to the intended dielet rather than the group of dielets.

**Figure 2:**
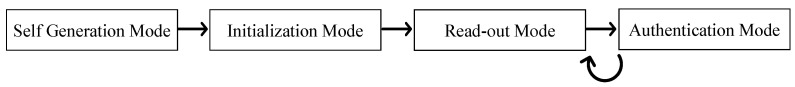
The CTR-SHIELD protocol comprises four modes. Following the self-generation and initialization modes, the dielet undergoes a repetition of the read-out and authentication modes.

**Figure 3:**
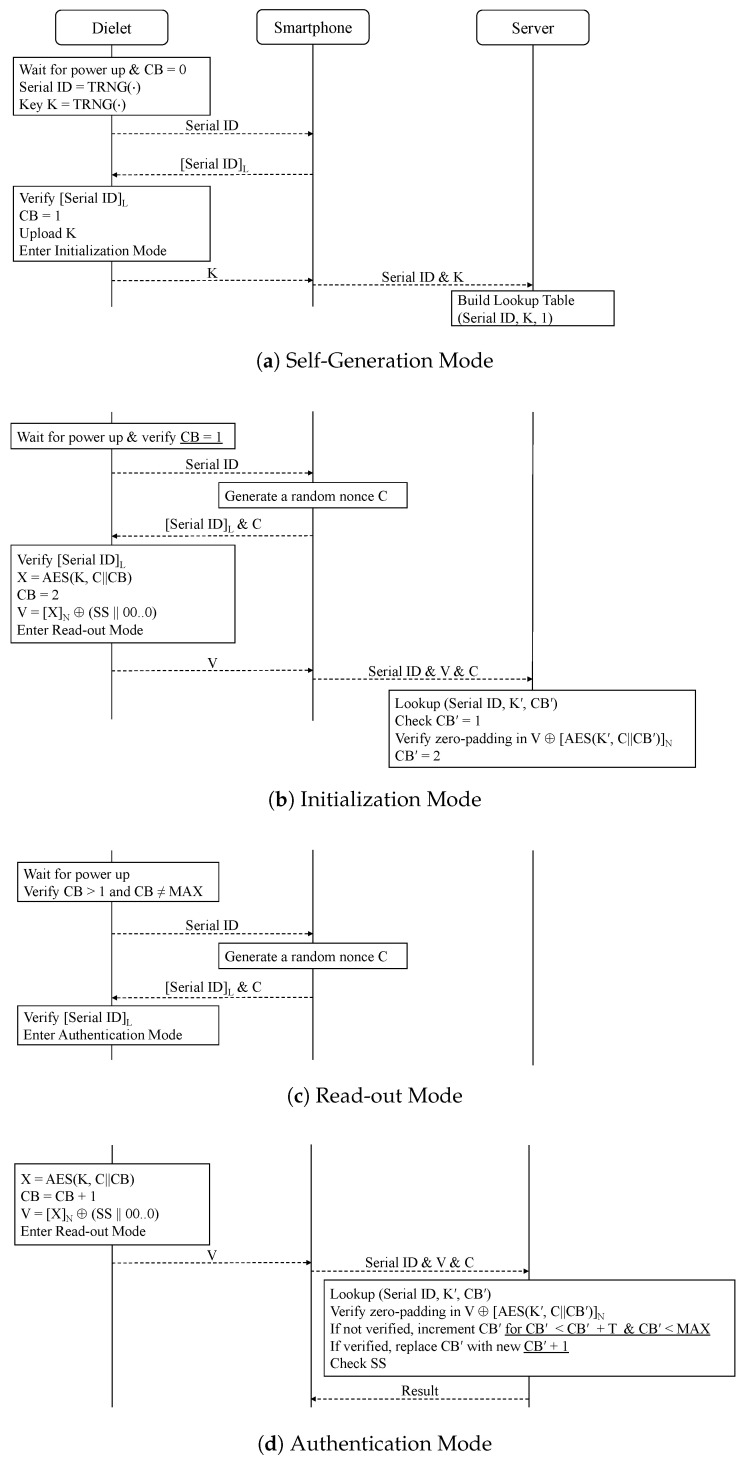
The CTR-SHIELD protocol, with an underlying assumption of a secure communication channel between the smartphone and the server. The underlined portions indicate our corrections.

Figure 2 illustrates the self-generation, initialization, read-out, and authentication modes of CTR-SHIELD. Using a powerful RF antenna, the manufacturer can simultaneously activate all dielets within a wafer. This simultaneous activation facilitates the concurrent generation of a key and random serial ID for each dielet, which are subsequently stored in NVM. The process of generating and storing these keys and IDs in the NVMs of the dielets takes only a few microseconds. After generating the keys and IDs, they are uploaded individually to the server as the dielets leave the wafer. The counter block *CB* is initialized to zero and set to one after the self-generation mode is complete, as demonstrated in Figure 3a.

After the generation and uploading of serial IDs and keys for all dielets on the wafer, they are made ready for transportation from the secure dielet fabrication facility. When the dielets arrive at the trusted IC assembly facility, they are embedded into the host package of legitimate ICs and undergo the initialization mode. This phase ensures the authentication of each dielet, activates the dielets’ passive sensors, and confirms the respective database entry for subsequent authentication outside the secure setting. Throughout this process, *CB* becomes two.

It should be noted that the authors in [25] states that each dielet performs one authentication session during this stage. The session begins by waiting for power up and verifying *CB* > 1 and *CB* ≠ *MAX*. However, in the initialization mode, the value of *CB* must be one, which means it cannot satisfy this condition. Therefore, the first authentication session should verify if *CB* = 1 and commence by transmitting the serial ID to the smartphone, as we have corrected in Figure 3b.

In the read-out mode depicted in Figure 3c, the dielet undergoes a verification process to ensure that its counter value, *CB*, satisfies specific conditions. It verifies whether *CB* is greater than one and not equal to *MAX*, representing the maximum acceptable number of times into the authentication mode for the dielet. If this verification fails, it indicates that the dielet is either not fully initialized yet (*CB* equals zero or one) and needs to proceed through the self-generation and initialization mode, or it has reached the *MAX* limit. On the other hand, if the verification succeeds, the dielet forwards its serial ID to the smartphone. The smartphone then truncates the serial ID to *L* bits and sends it back to the dielet, accompanied by a uniformly distributed *M*-bit random nonce, denoted as *C*. The dielet will only enter the authentication mode if the truncated serial ID is accurate. However, if the truncated serial ID is incorrect, the dielet will refrain from entering the authentication mode as this could potentially indicate a batch mode of DoS attacks.

In the authentication mode shown in Figure 3d, the key *K* encrypts the concatenation of *C* and *CB* to produce a ciphertext *X*. Subsequently, *CB* is incremented, and the dielet performs an XOR operation on the first *N* bits of *X* with its passive sensor status bits (SS), extended with zeroes, to create an *N*-bit verification message *V*. After this, the dielet returns to the read-out mode and waits for the next authentication request. The smartphone sends *V*, along with the serial ID and random nonce *C*, to the server. Utilizing these inputs, the server calculates $X^{\prime }$ and applies an XOR operation to the leading *N* bits of $X^{\prime }$ with *V* to derive a result *Z*. If *Z* reveals the existence of the padded zero bits, it confirms that the dielet linked to the serial ID generated the ciphertext. Moreover, the initial *S* non-zero bits of *Z* are interpreted as the dielet’s passive sensor status bits. If the authentication fails, the server increments ${\mathrm{CB}}^{\prime }$ and repeats the process unless the authentication is successful, with the condition that the number of increments is less than the predetermined threshold *T*, or ${\mathrm{CB}}^{\prime }$ is less than *MAX*.

The authors in [25] presented a figure of their authentication protocol, which illustrates that the server increments ${\mathrm{CB}}^{\prime }$ up to *T* times, with a maximum value of ${\mathrm{CB}}^{\prime }=\mathrm{MAX}$ if authentication fails. However, this description is inaccurate because in the read-out mode, the dielet first checks whether CB≠MAX. Therefore, a legitimate dielet cannot have the value CB=MAX. Furthermore, *CB* is incremented up to *T* − 1 times rather than up to *T* times. The correct illustration of their method is shown in Figure 3d, where we underline to indicate that ${\mathrm{CB}}^{\prime }$ is incremented up to *T* − 1 times, with a maximum value of *MAX*-1.

Here, we provide another correction regarding the authentication mode in CTR-SHIELD. Previously, it was stated that ${\mathrm{CB}}^{\prime }$ is updated to a new value (${\mathrm{CB}}^{\prime }$) when the zero padding is verified. However, this is incorrect because the dielet’s CB is actually incremented by one in the authentication mode. In Figure 3d, we underline the relevant section to highlight our correction regarding the update of ${\mathrm{CB}}^{\prime }$ (= ${\mathrm{CB}}^{\prime }$ + 1), which ensures the exact synchronization of counters between the dielet and the server.

While the protocol from DARPA, illustrated in Figure 1, requires two full rounds of communication between the smartphone and the remote server, the CTR-SHIELD protocol reduces the communication rounds to just one. This results in a doubling of the communication speed compared with the DARPA protocol. Additionally, the dielet efficiently reduces the required number of encryptions from two to one, eliminating the need to verify server messages during the read-out and authentication modes.

However, this improvement comes with a drawback in the form of a security vulnerability to DoS attacks. The simple act of incrementing the dielet’s counter by confirming the truncated serial ID from any smartphone exposes a vulnerability that malicious attackers can exploit to disrupt the availability of the dielet through synchronization issues. Consequently, CTR-SHIELD’s read-out mode can only withstand batch-mode desynchronization attacks, making it ineffective against attacks targeting a single dielet. If an attacker can persistently desynchronize each dielet from a remote location by sending the truncated serial ID, they can easily desynchronize an entire group of dielets. Due to this limitation, the read-out mode does not offer significantly enhanced protection against DoS attacks.

## 3. Proposed Scheme

In this section, we introduce our enhanced authentication protocol aimed at offering improved defense against DoS attacks, specifically desynchronization attacks. To accomplish this, we incorporate a challenge–response pair and involve the server in the read-out mode, similar to DARPA’s protocol. Table 1 presents the key notations newly introduced in our proposed protocol.

To authenticate the response from the server, the dielet requires additional encryption. However, if there were network connection issues during the previous communication, the counters may become desynchronized. This would result in multiple encryption operations if the dielet attempted to synchronize the counters by backtracking the counter block (*CB*).

To address this and reduce computational costs, our protocol incorporates an additional counter block called the check point (*CP*). The proposed protocol employs two distinct counter blocks with specific purposes. *CB* is used to limit the maximum number of times the dielet can enter authentication mode, preventing counter rollover. On the other hand, *CP* stores the value of the server’s counter block that most recently succeeded in authentication at the dielet’s end.

By leveraging *CP*, the dielet avoids the need for multiple encryption operations to search for the counter value that satisfies the server’s challenge–response pair. Due to these advancements, we refer to our protocol as DTR-SHIELD, where DTR refers to double counter blocks. Further details on the proposed protocol are provided below.

### 3.1. Self-Generation and Initialization Modes

Our protocol aims to prevent desynchronization attacks by malicious attackers through the server generating and transmitting random challenges. To prevent replay attacks carried out by man-in-the-middle attackers, both the server and the client maintain a list, denoted as *H* (which stands for history), which stores the first *R* bits of previously utilized challenges. When a new dielet record is inserted into the database, an empty list *H* with *B* (which stands for backup) elements is generated during the self-generation mode.

Our system utilizes the initialization mode of the CTR-SHIELD protocol. In this mode, upon receiving the serial ID from the dielet, the smartphone generates a random challenge *C*. The truncated serial ID and *C* are then sent back to the dielet. Using the AES algorithm, the concatenated block of *C* and *CB* is encrypted with the secret key *K*, resulting in the ciphertext *X*. To reduce the message length, *X* is truncated to *N* bits and is represented as ${\left[X\right]}_{N}$. To derive the value *V*, the sensor status bits have an XOR operation performed on them with ${\left[X\right]}_{N}$, and zero padding is appended to the result. The counter *CB* is incremented to two, and the dielet enters the read-out mode. In addition to the above, our initialization mode incorporates *CP*. This counter is used to store the most recent value of *CB* that successfully verifies the challenge and response pair sent by the server during the read-out mode. In the initialization mode, *CP* is also set to two to ensure proper operation.

Upon receiving the serial ID, *V*, and *C* from the smartphone, the server first checks if the current value of *CB*′ is one. If it is, the server generates $X^{\prime }$ using the same challenge *C* and its counter *CB*′. To verify the zero padding, the server XORs ${\left[X\right]}_{N}^{\prime }$ with *V*. If the zero padding is validated, the counter *CB*′ stored on the server side is incremented to two.

### 3.2. Read-Out Mode

When the dielet is in read-out mode, it remains inactive until it receives power. Upon powering up, the dielet verifies the value of the counter *CB* to ensure that it falls within the acceptable range of one to *MAX*. If the check fails, it indicates that the dielet needs to enter the self-generation and initialization mode, as explained earlier. Alternatively, the dielet might have exceeded the acceptable number of attempts to enter the authentication mode, in which its sensor status is read, encrypted, and sent to the server. Keeping *CB* below the *MAX* value limits the encryption by the dielet and prevents counter rollover, averting a counter reset.

Once the verification is successful, the dielet sends its serial ID to the server via the smartphone. The server retrieves the dielet’s record and generates two *M*-bit random challenges, denoted as C1 and C2, where ${\left[C1\right]}_{R}\notin H$. In *H*, the first *R* bits of *B* previously used C1s are stored. When the length of C1, denoted as *M*, is significantly larger than *R*, if the server generates C1 randomly, it can produce a new C1 with the first *R* bits not included in *H*. The server then encrypts the concatenated block of C1 and *CB*′ to obtain $D^{\prime }$ and sends [serial ID]_*L*_, C1, C2, and $D^{\prime }$ to the dielet.

Before verifying the server’s reply, the dielet performs three checks. First, it compares the truncated serial ID received with its own to ensure they match. Second, it checks whether the value of C1 used in the reply has not been recently used by verifying that ${\left[C1\right]}_{R}\notin H$. This prevents a reply attack using a previously sent message from the server. If both conditions are met, the dielet checks if the difference between *CB* and *CP* is not greater than *T*. If the difference exceeds *T*, it indicates that the network connection is not in normal condition or has been disrupted by malicious attackers. As a result, the dielet does not proceed to the next computation of *D*; instead, it generates a random nonce. Without entering the authentication mode, the dielet transmits this nonce as if it was *V*.

If the difference is not greater than *T*, the dielet encrypts the concatenated block of C1 and *CB* to obtain *D*. If $D=D^{\prime }$, the dielet enters the authentication mode and adds ${\left[C1\right]}_{R}$ to *H*. Additionally, *CP* is set to *CB*, reflecting the most recent value that satisfies the verification of $D^{\prime }$ sent by the server.

If *D* does not match $D^{\prime }$, it may indicate desynchronized counters where $\mathrm{CB}>{\mathrm{CB}}^{\prime }$. This discrepancy can occur when the dielet increments CB even in cases of message loss during the authentication mode. As a result, $\mathrm{CB}\geq {\mathrm{CB}}^{\prime }$. To address this issue, the dielet now utilizes *CP* to compute a new *D* and compares it with $D^{\prime }$. If the comparison still results in a mismatch, the dielet will not proceed to the authentication mode. This is because, in such a scenario, only *CP* represents a valid value for the server’s ${\mathrm{CB}}^{\prime }$. To prevent the disclosure of the success or failure of the read-out check, the dielet will transmit a random number instead of the expected message *V*.

### 3.3. Authentication Mode

In the read-out mode, the synchronization between the counters shared by the dielet and the server is verified to detect any possible malicious desynchronization attack. If the difference falls within acceptable limits, the authentication mode starts by concatenating the second challenge C2 and *CB*, which is then encrypted to generate the block *X*. This block is truncated to [*X*]_*N*_, and *V* is obtained by XORing [*X*]_*N*_ with the sensor status bits *SS*, padded with zeros. The counter block *CB* is incremented by one, and the resulting *V* and dielet’s serial ID are sent to the server. After transmitting this information, the dielet reverts to read-out mode.

To authenticate a dielet, the authentication server retrieves the key $K^{\prime }$ and the counter block *CB*′ corresponding to the dielet’s serial ID. Using these values, the server calculates $X^{\prime }$ = *AES*($K^{\prime }$, C2 || *CB*′), where C2 is the second challenge. The first *N* bits of $X^{\prime }$ are XORed with *V*. If the outcome matches the expected pattern of *SS* padded with zeros, the server concludes that the dielet associated with the serial ID produced the ciphertext. Moreover, the leading *S* non-zero bits of *Z* are interpreted as the sensor status bits.

However, connectivity issues such as server disconnection can lead to troublesome desynchronization between *CB* and *CB*′. To address this problem, the authentication server employs a solution where it incrementally increases *CB*′ and re-executes the authentication protocol until authentication succeeds (updating *CB*′ in the lookup table) or the number of increments surpasses a predefined threshold, denoted as *T*. If authentication fails *T* times, it indicates a single-dielet desynchronization attack, and the authentication permanently fails for that specific dielet, aligning with SHIELD’s principles, which mandate discarding compromised dielets.

**Figure 4:**
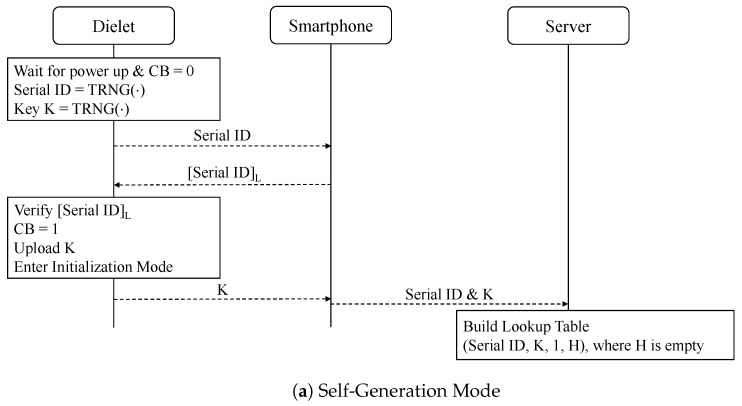

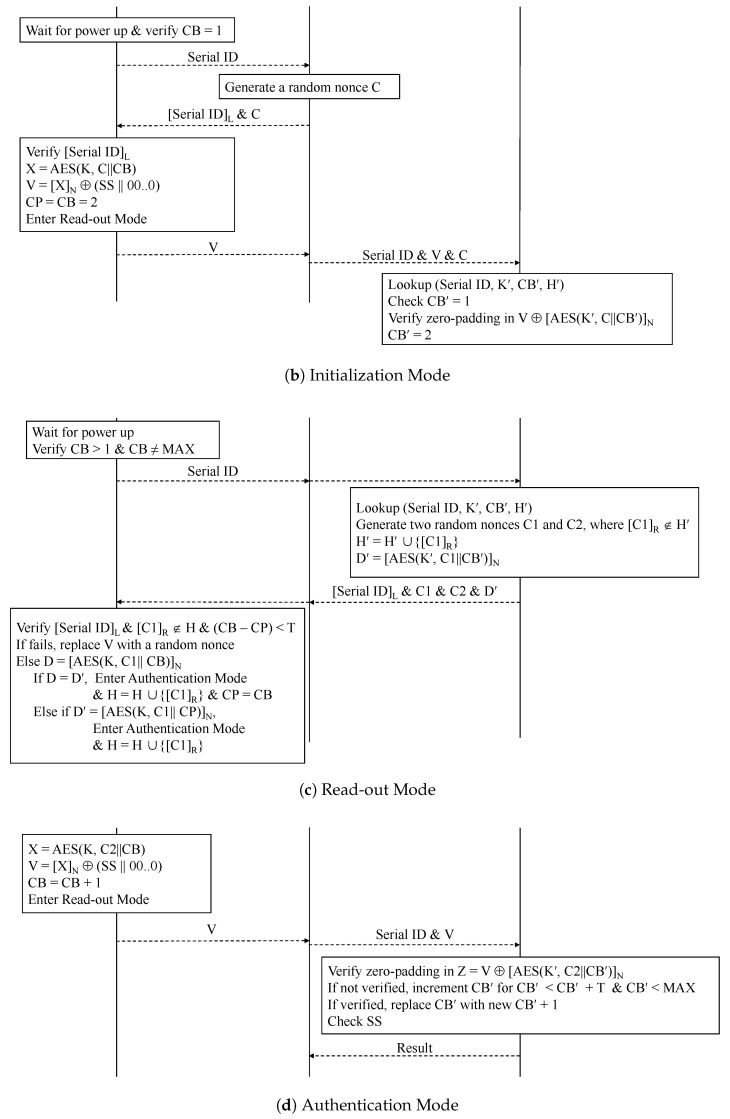
The proposed DTR-SHIELD protocol. It assumes a secure communication channel between the smartphone and the server.

This approach utilizes the counter block to detect single-dielet desynchronization attacks, obviating the need for costly recovery procedures. All *T* attempts take place solely on the server and do not require additional communication between the dielet and the server, minimizing performance overhead. Once authentication is successfully completed, including the verification of zero padding and sensor status bits, the authentication result is transmitted to the smartphone. The complete authentication protocol is illustrated in Figure 4.

## 4. Evaluation

### 4.1. Security

In this subsection, the evaluation of protection against desynchronization attacks is the main focus. Prior to that, the trust model and threat model for DTR-SHIELD are introduced, which are identical to those utilized in CTR-SHIELD.

**The trust model:** The trust model for DTR-SHIELD revolves around the integration of trustworthy dielets into host packages within the IC supply chain. Trust is crucial at multiple stages from dielet design to initialization, with a presumption of secure transit from dielet design to fabrication. Dielets possess the capability to verify the identity and authenticity of chips at any point in the supply chain. For secure verification and the prevention of malware attacks, smartphones need to be trusted. A viable approach involves leveraging a trusted execution environment, such as ARM TrustZone. It is assumed that the communication channel between the smartphone and the server is secure. To enhance protection against vulnerabilities in the supply chain, DTR-SHIELD can be integrated with additional security measures and testing approaches. However, it is important to note that DTR-SHIELD does not aim to address security issues that may arise in chip design, fabrication, and assembly facilities.
**Threat model:** We mainly considers two primary types of attacks: DoS and impersonation attacks. DoS attacks, also referred to as desynchronization attacks in this paper, aim to disrupt the proper functioning of legitimate ICs and can be executed individually or in groups (*DA*). On the other hand, impersonation attacks involve the insertion of counterfeit or malicious chips into the supply chain. Impersonation attackers may possess valid serial IDs (*IA1*), have oracle access to legitimate dielets (*IA2*), be able to separate dielets from host chips and reuse them (*IA3*), or have the capability to extract secret keys from dielets through physical attacks (*IA4*).

In order to assess the security of the proposed protocol, we will examine the potential attacks outlined in the threat models as follows. It is worth noting that the security against *IA1*–*IA4* is inherited from CTR-SHIELD, as DTR-SHIELD improves the read-out and authentication modes to defend against single-mode desynchronization attacks.

***DA*** **Security:** In CTR-SHIELD, the read-out mode switches to authentication mode once the first *L* bits of a dielet’s serial ID are verified, and the counter *CB* is incremented on the dielet side. This creates a vulnerability where a malicious smartphone can desynchronize *CB* by replying with truncated serial IDs.

To tackle this problem, our read-out mode includes supplementary verification steps (*CB* − *CP*) < *T* to ensure the synchronization of *CB* and *CB*′, where CB∈ {*CB*′, *CB*′ − 1, *…*, *CB*′ − T − 1}. To test the equality between the counters, we use the server’s challenge C1 for computing *D*, which has not been used recently.

A successful attack cannot be executed solely through eavesdropping and retransmitting past messages. To increase their chances of success, the attacker must choose an *M*-bit C1 such that ${\left[C1\right]}_{R}$ is not found in *H* with a probability of 1 − $B/2^{R}$. In such situations, the probability of accurately guessing a valid C1 and the correct message $D^{\prime }$ is approximately

$$\left(1-B/2^{R}\right)\times 2/2^{N}.$$

This is because the dielet performs computations for *D* up to two times: once with *CB* and another encryption with *CP* if *CB* is not satisfied. This assumes a realistic scenario where the attacker cannot eavesdrop on the communication between the legitimate dielet and smartphone to the extent that they can perform replay attacks using legitimate (C1, $D^{\prime }$) pairs that were used before successful authentication with *B* legitimate pairs. Considering this assumption, the attacker’s advantage heavily relies on the size of *N* in comparison with other parameters like *B* and *R*.

To achieve successful desynchronization of the counters, the attacker needs to choose a valid pair of (C1, $D^{\prime }$) *T* times. In summary, the probability of successful *DA* is given by

$$\leq {\left(\left(1-B/2^{R}\right)\times 2/2^{N}\right)}^{T}={\left(2/2^{N}-2B/2^{NR}\right)}^{T}.$$

This is because the server attempts to synchronize with a *T*-time decrement on *CB*′ while considering desynchronized counters. However, the attacker cannot determine the success of each pair due to the random nonce sent in response when verification fails. Considering the limited time available for a potential attacker to access a legitimate dielet, the probability of a successful desynchronization attack is low.

***IA1*** **Security:** An *IA1* attacker, represented by A, aims to create a counterfeit dielet F using only the current list of validated serial IDs, denoted by L, from the server. The fake dielet F must satisfy the verification protocol to remain undetected. By the initialization protocol’s definition, the generation of L is entirely isolated from key generation. Additionally, L does not reveal any plaintext–ciphertext pairs. Therefore, understanding L only serves to aid A in choosing a legitimate serial ID for F.

To pass the verification process depicted in Figure 4, A must fabricate a counterfeit dielet F that generates a valid message V=[AES(K,C2||${CB)]}_{N}⊕\left(\mathrm{SS}||00\ldots 0\right)$, where SS= “OK” for a randomly chosen challenge C2 and a key *K* corresponding to the ID. As the value (SS||00…0) remains constant, the objective of A is to construct a fake dielet F that only computes the leading *N* bits of the ciphertext AES(K,C2||*CB*), where C2 is a random challenge generated by the server, *CB* ∈ {*CB*′, …, *CB*′ +T−1}, and *K* and ${\mathrm{CB}}^{\prime }$ match the ID in the server’s database. Although A can fulfill the roles of both the dielet and smartphone during the authentication protocol, the randomness of C2 prevents A from gaining any knowledge on how to create such a F. Here, the uniform distribution of true randomness implies that the chance of guessing one correct output is precisely $1/2^{N}$. In this scenario, A has a probability of approximately $T/2^{N}$ of generating a valid response *V*.

***IA2*** **Security:** Based on the definition of the initialization protocol, it is evident that each secret key is generated independently. This implies that if an adversary A has oracle access to multiple legitimate dielets $\{D_{j}|j\neq i$ and i,j<m}, they will not gain any information about dielet $D_{i}$.

To create the fake dielet F, A needs to generate valid challenge–response pairs due to the modified read-out mode. Let us consider oracle access to $D_{i}$, which has a serial ID used by F. In order to collect challenge–response pairs $\left(C2_{j},V_{j}\right)$, where $V_{j}=[AES(K,C2_{j}||CB_{j}{)]}_{N}⊕\left(\mathrm{SS}||00\ldots 0\right)$ and each counter block ${\mathrm{CB}}_{j}$ is unique, A must first generate a valid $D^{\prime }$ with an unused challenge C1 without knowing the value of *K*. The probability of success is $\left(1-B/2^{R}\right)\times 2/2^{N}$, as previously explained. After making *q* queries, the probability of collecting *q* valid pairs is negligible, approximated by ${\left(\left(1-B/2^{R}\right)\times 2/2^{N}\right)}^{q}$.

During an authentication session, F can successfully impersonate a legitimate dielet by responding with the corresponding verification message $V_{h}$ if the random nonce C2 provided by the server matches one of the challenges $C_{h}$, and the counter value ${\mathrm{CB}}_{h}$ utilized in generating ($C_{h},V_{h}$) falls within the permissible range of counter values {${\mathrm{CB}}^{\prime },\ldots ,{\mathrm{CB}}^{\prime }+T-1$}, where no ${\mathrm{CB}}_{j}$ repeats. The probability of C2 colliding with one of the ≤T challenges $C_{j}$ is $\leq T/2^{M}$, given that C2 is randomly selected from $2^{M}$ possible bit strings. If C2 does not match any of these ≤T challenges $C_{j}$, the probability of generating a correct ciphertext ${\left[AES(K,C2||CB)\right]}_{N}$ for a $CB\in \{{\mathrm{CB}}^{\prime },\ldots ,{\mathrm{CB}}^{\prime }+T-1\}$ is approximately $T/2^{N}$. Therefore, the advantage of the *IA2* attacker can be estimated as approximately

$${\left(\left(1-B/2^{R}\right)\times 2/2^{N}\right)}^{q}\times \left(T/2^{M}+T/2^{N}\right).$$

***IA3 and IA4*** **Security:** An *IA3* attack A can remove a legitimate dielet from its package and attach it to a counterfeit chip. This process may trigger passive sensors with probability ρ, which affects the verification message in authentication mode:

$$V={\left[AES(K,C2||CB)\right]}_{N}⊕\left(\mathrm{SS}||00\ldots 0\right),$$

where SS equals “OK” with probability 1−ρ and is not equal to “OK” with probability ρ. Because ρ>1−ρ, the most likely hypothesis is that *V* corresponds to SS≠ “OK”. To launch a customized *try-and-check* attack against DTR-SHIELD, the attacker must generate a valid $D^{\prime }$ with a recently unused challenge C1 without knowing the value of *K*. The probability of success is negligible, as previously analyzed. The attacker’s next challenge is to distinguish between a truncated AES ciphertext and a random nonce in authentication mode to determine whether the passive sensor has detected the dielet detachment. However, the attacker has a negligible advantage because the AES output becomes indistinguishable from random after the third round [30].

Moreover, limiting the value of *MAX* can thwart *IA4*’s power analysis [31,32] as the attacker cannot gather sufficient power traces. Thus, power analysis has a low likelihood of success in the presence of measurement noise. Furthermore, to perform differential fault analysis [33,34,35,36], the attacker needs to disable the counter’s increment to obtain valid differential equations. However, this is not easy to achieve, and a passive sensor may detect the fault injection attempt. As discussed in CTR-SHIELD, this solution remains vulnerable to probing attacks [37], similar to other existing solutions.

### 4.2. Performance and Costs

With the given parameters, namely *L* = 30, *N* = 50, *M* = 50, and *T* = 8, like in the case of CTR-SHIELD, DTR-SHIELD introduces two supplementary parameters, *B* = 5 and *R* = 10. The size of *H* is only 50 bits in total. The rationale behind selecting small values for *B* is rooted in our previous explanation: we assume that it is improbable for an attacker to maintain close proximity to a legitimate dielet for an extended period, which is necessary for intercepting a substantial volume of communication between the target dielet and smartphone. Within this configuration, DTR-SHIELD incurs additional costs, as delineated below. It is important to note that certain minor costs, such as bit concatenation, range checking, and set operations on *H*, are not factored into our analysis.

- In our protocol, each authentication request generally involves performing double encryption, whereas CTR-SHIELD only requires a single encryption. If the counter blocks *CB* and *CB*′ become desynchronized, our protocol requires an additional encryption to verify the server’s involvement in computing the response. Consequently, the dielet experiences two to three times the power consumption associated with AES encryption. This power consumption is comparable with DARPA’s SHIELD protocol, which also employs double encryption. In the case of authentication failure, a TRNG is used to generate a random nonce instead of computing a valid message *V*. Additionally, *H* occupies R×B bits (which equals 50 for the specified parameters) of non-volatile memory space in the dielets. In addition to the 128-bit secret key and an 8-bit counter block, an extra 8-bit block must also be stored.
- For a 128-bit serial ID, our solution transmits more bits in the read-out mode between the dielet and the smartphone compared with CTR-SHIELD. Specifically, the total number of transmitted bits between the dielet and the smartphone is calculated as 128 + *L* + 2M + 2N. In contrast, CTR-SHIELD transmits 128 + *L* + *M* + *N*. Therefore, DTR-SHIELD requires additional *M* + *N* bits to be transmitted. Given the specified parameters, the extra transmission amounts to 100 bits.
- In the read-out and authentication modes of our protocol, communication between the smartphone and the server consists of two rounds, while CTR-SHIELD requires just one complete round. However, the initial round of communication in our protocol is crucial to protect against desynchronization attacks. It is important to highlight that during this first round, the server performs the initial encryption to compute $D^{\prime }$, which might introduce some latency. For this reason, under normal circumstances, the server performs two encryptions in both the read-out and authentication modes, while CTR-SHIELD requires only one encryption for both modes.

In summary, Table 2 presents a concise comparison of the security and performance characteristics between the two previous SHIELD protocols and our proposed DTR-SHIELD. The performance comparison assumes that the parameters presented in this section are used. As the number of dielets communicating with a smartphone increases, and assuming no desynchronization issues, each dielet performs one additional encryption, requires 100 extra bits to be transmitted between the dielet and the smartphone, and involves one more encryption at the server side. The following cost analysis estimates the implementation and computational costs.

According to [25], the CTR-SHIELD protocol was implemented using 32 nm technology. This implementation included an SRAM-based TRNG with a von Neumann extractor, an 8-bit counter, and additional control logic. A state machine was developed to manage the four operational modes of the dielet, and its control logic was compared with DARPA’s protocol. To optimize the dielet’s area, operations were performed byte-wise using a compact 8-bit datapath AES-256 encryption core, which required 224 clock cycles per encryption. The AES engine employed an S-box implementation that transformed input values across composite fields, ensuring efficiency and minimal area usage for over a decade. In our DTR-SHIELD implementation, the AES-256 circuit occupies the same area as in CTR-SHIELD but requires an additional 244 or 488 clock cycles for AES encryption. The AES-256 core occupied 55% of the dielet’s 0.01 mm^2^ area, while the control logic required 6%, compared with 2% in DARPA’s protocol. For CTR-SHIELD, the extra area for a 64-bit NVM, necessary for storing a 128-bit serial ID, was negligible (<0.01 $\;{\mathrm{mm}}^{2}$) in 32 nm technology. Similarly, DTR-SHIELD, which requires an additional 58-bit NVM compared with CTR-SHIELD, also has a negligible area overhead (<0.01$\;{\mathrm{mm}}^{2}$).

## 5. Conclusions and Future Work

To address the risks posed by outsourcing IC fabrication due to semiconductor production globalization, DARPA proposed the SHIELD program, which incorporates a dielet as a secure hardware root-of-trust into ICs, aiming to enhance security. Dielets are RF-powered devices that passively record malicious events. The CTR-SHIELD protocol provides a framework for initialization and authentication protocols, introducing various adversarial models. It addresses a specific “try-and-check” attack and presents an improved authentication protocol. However, the protocol has a critical vulnerability to desynchronization attacks, jeopardizing the effectiveness of legitimate dielets in the SHIELD program’s security.

In this paper, we introduce DTR-SHIELD as an enhanced solution to address the vulnerability of desynchronization attacks in the CTR-SHIELD protocol. The root cause of desynchronization lies in the simplicity of counter incrementation by responding with a truncated serial ID alone. To overcome this issue, we propose modifications to the initial, read-out, and authentication modes to ensure the legitimate server’s active involvement in the communication process.

In the modified read-out mode, a crucial requirement for incrementing the dielet’s counter block is a challenge and response pair from the server. If the verification process fails, the dielet performs an additional verification by checking if the challenge can generate the response using the recently verified counter. In the event that both counters fail to verify the server’s response, the dielet responds with a random nonce instead of an authentication message. This approach effectively safeguards the shared counters against desynchronization. Overall, our protocol introduces an additional AES encryption and necessitates the transmission of 100 extra bits from the dielet compared with CTR-SHIELD. These modifications significantly enhance the defense against desynchronization attacks.

Our future work can be summarized as follows: Firstly, we aim to decrease the number of encryption operations, thereby reducing the implementation costs associated with the chips. Secondly, we seek to minimize the communication cost between the dielet and the smartphone to enhance the protocol’s efficiency. Lastly, we plan to validate the proposed approach through the design and implementation of a dielet with PUF-based TRNG.

## Figures and Tables

**Table 1:** Notations.

Notation	Description
*CB*	Counter block for limiting the maximum number of dielet’s authentications
*CP*	Counter block for tracking server’s counter block at the dielet
*H*	List of previously used challenges
*B*	The number of challenges stored in *H*
*C*1 and *C*2	Sever’s two random challenges
*D*	Encrypted data of C1 and either *CB* or *CP*
*X*	Encrypted data of C2 and *CB*

**Table 2:** Comparison between the previous SHIELD protocols and ours in terms of security and performance. O: secure; X: vulnerable.

	Security		Performance		
	**Try-and-Check Attack**	**Single-Dielet DoS Attack**	# **of AES Encryption**	**Comm. between Dielet and Smartphone**	**Memory**
SHIELD [24]	X	O	2	448 bits	192 bits
CTR-SHIELD [25]	O	X	1	158 bits	264 bits
DTR-SHIELD	O	O	2 or 3	258 bits	322 bits

## Data Availability

The data are contained within the article.
